# Role of Oxidative Stress in Metabolic and Subcellular Abnormalities in Diabetic Cardiomyopathy

**DOI:** 10.3390/ijms21072413

**Published:** 2020-03-31

**Authors:** Naranjan S. Dhalla, Anureet K. Shah, Paramjit S. Tappia

**Affiliations:** 1 Institute of Cardiovascular Sciences, St. Boniface Hospital Albrechtsen Research Centre, Winnipeg, MB R2H 2A6, Canada; 2 Department of Physiology and Pathophysiology, College of Medicine, Rady Faculty of Health Sciences, University of Manitoba, Winnipeg, MB R3E 0J9, Canada; 3 Department of Kinesiology, Nutrition and Food Science, California State University, Los Angeles, CA 90032, USA; akaur23@calstatela.edu; 4 Asper Clinical Research Institute, St. Boniface Hospital, Winnipeg, MB R2H 2A6, Canada; ptappia@sbrc.ca

**Keywords:** sympathetic nervous system, renin-angiotensin system, oxidative stress, subcellular remodeling, Ca^2+^-handling abnormalities, cardiac metabolism, diabetic cardiomyopathy

## Abstract

Although the presence of cardiac dysfunction and cardiomyopathy in chronic diabetes has been recognized, the pathophysiology of diabetes-induced metabolic and subcellular changes as well as the therapeutic approaches for the prevention of diabetic cardiomyopathy are not fully understood. Cardiac dysfunction in chronic diabetes has been shown to be associated with Ca^2+^-handling abnormalities, increase in the availability of intracellular free Ca^2+^ and impaired sensitivity of myofibrils to Ca^2+^. Metabolic derangements, including depressed high-energy phosphate stores due to insulin deficiency or insulin resistance, as well as hormone imbalance and ultrastructural alterations, are also known to occur in the diabetic heart. It is pointed out that the activation of the sympathetic nervous system and renin–angiotensin system generates oxidative stress, which produces defects in subcellular organelles including sarcolemma, sarcoplasmic reticulum and myofibrils. Such subcellular remodeling plays a critical role in the pathogenesis of diabetic cardiomyopathy. In fact, blockade of the effects of neurohormonal systems has been observed to attenuate oxidative stress and occurrence of subcellular remodeling as well as metabolic abnormalities in the diabetic heart. This review is intended to describe some of the subcellular and metabolic changes that result in cardiac dysfunction in chronic diabetes. In addition, the therapeutic values of some pharmacological, metabolic and antioxidant interventions will be discussed. It is proposed that a combination therapy employing some metabolic agents or antioxidants with insulin may constitute an efficacious approach for the prevention of diabetic cardiomyopathy.

## 1. Introduction

Type 1 diabetes occurs as a result of insulin deficiency due to a defect in insulin-producing β-cells in the islets of Langerhans of the pancreas, while type 2 diabetes occurs as a consequence of insulin resistance/insensitivity of insulin receptors [1]. In 2019, the World Health Organization reported that the global diabetic population increased from 108 million in 1980 to 422 million in 2014. The diabetic population is at increased predisposition to developing different complications including microangiopathy, atherosclerosis, vascular disease and heart failure [2,3]. In fact, diabetics have a more than 2-fold higher risk for developing heart failure independent of other co-morbidities [4,5]. Although diabetes-induced cardiac complications were considered to be due to myocardial ischemia as a consequence of the development of atherosclerosis in coronary arteries, the pioneering work of Timothy Regan suggested the existence of diabetic cardiomyopathy, particularly when diabetic patients presented with a reduced left ventricular ejection fraction, an increase in pre-ejection time and increased end-diastolic pressure [1,2]. These cardiac functional changes, in the absence of coronary artery disease, were considered to be due to increases in ventricular wall stiffness and isovolumic relaxation as well as depressed cardiac contractility [4,5]. 

**Figure 1:**
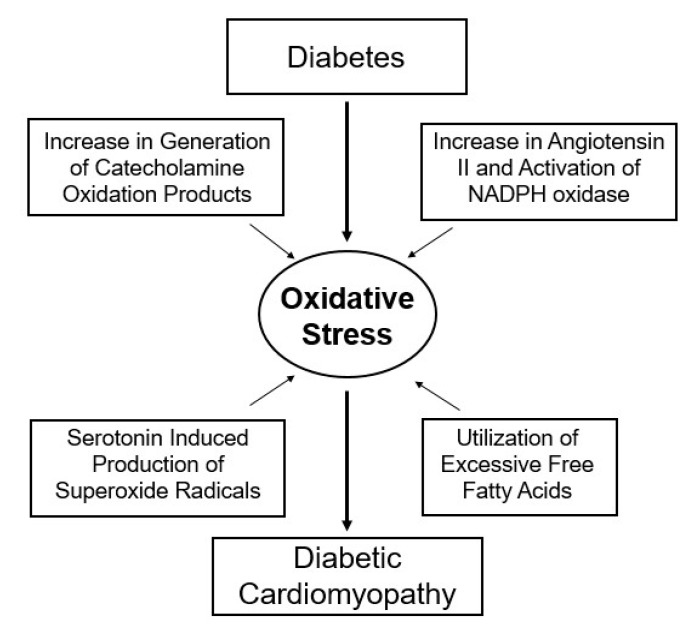
Role of oxidative stress in the pathogenesis of diabetic cardiomyopathy.

Diabetes has also been shown to be associated with the activation of sympathetic nervous system (SNS) and increased outflow of catecholamines, and the activation of the renin–angiotensin system (RAS) and increased formation of angiotensin II. The elevated levels of these vasoactive hormones due to insulin-deficiency are considered to produce marked defects in myocardial metabolism and remodeling of sarcolemma (SL), sarcoplasmic reticulum (SR), mitochondria (MT) and myofibril (MF) [6,7,8,9,10,11,12,13]. It is now well established that oxidative stress occurs as a consequence of an imbalance between reactive oxygen species (ROS) production and endogenous as well as exogenous oxidant scavenging systems [14]. Several lines of experimental and clinical evidence have demonstrated that ROS, such as superoxide anion, hydroxyl radicals and hydrogen peroxide, mediate oxidative actions in the pathogenesis of diabetes-related cardiovascular complications [15,16,17,18,19,20]. It should be noted that hyperglycemia-induced increase in glucose autooxidation, protein glycation and oxidative degradation of glycated proteins leads to an excessive generation of ROS under diabetic conditions [21,22,23]. Furthermore, since hyperglycemia, hormone imbalance and a shift in myocardial metabolism result in ROS overproduction, oxidative stress has been suggested to play an important role in subcellular remodeling and heart dysfunction in diabetes [9,10,11,12] (Figure 1). It is pointed out that while type I diabetes is generally characterized by hypoinsulinemia, type II diabetes is characterized by hyperinsulinemia or insulin resistance. However, both of these forms of experimental diabetes have been shown to exhibit hyperglycemia and hyperlipidemia, as well as cardiac dysfunction and subcellular remodeling that simulate the human condition [6,7,13,24,25,26,27,28]. 

In order to demonstrate that cardiac dysfunction in diabetes occurs progressively, and to examine the relationship between heart dysfunction and subcellular abnormalities, we have previously reported the time-course of changes in subcellular remodeling following the induction of diabetes in two well-established experimental models of type 1 diabetes using streptozotocin (STZ) and alloxan [29,30,31]. These agents are widely regarded as the most prominent cytotoxic diabetogenic glucose analogues for inducing experimental diabetes, while alloxan generates oxidative damage of insulin-producing β-cells, STZ causes DNA fragmentation and destroys the β-cells. It can be seen from Table 1 that maximal levels of plasma glucose and minimal levels of insulin become apparent in one week of administration of alloxan, whereas similar changes in the plasma glucose and insulin became evident within two to three weeks after STZ injection. It is also pointed out that metabolic, subcellular and functional alterations in the heart in both alloxan and STZ-induced diabetic models were markedly attenuated by insulin treatment [29,30,31].

**Figure 2:**
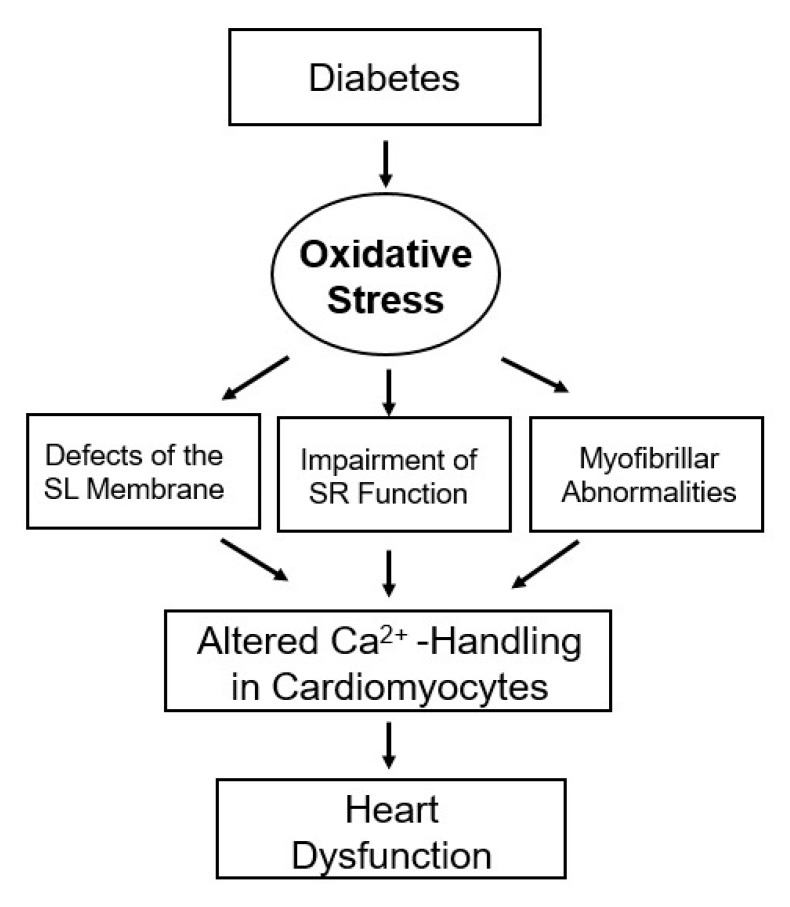
Role of oxidative stress in subcellular remodeling and Ca^2+^-handling abnormalities in the diabetic heart.

Table 1 shows varying degrees of depressions in SL Na^+^-K^+^-ATPase, SL Na^+^-Ca^2+^-exchanger, SR Ca^2+^-stimulated ATPase, SR Ca^2+^-uptake and MF Ca^2+^-stimulated ATPase was evident at 2 wks, whereas changes in cardiac function (LVEDP and LVSP), heart rate and mean arterial pressure were apparent at 4 wks after alloxan-induced diabetes [29,30]. Furthermore, the data presented in Table 2 [31] show that the increase in LVEDP and reduction in LVSP as well as depressions in SL Na^+^-K^+^-ATPase, SL Na^+^-Ca^2+^-exchanger, SR Ca^2+^-stimulated ATPase, SR Ca^2+^-uptake and MF Ca^2+^-stimulated ATPase were seen at 3 wks after the induction of diabetes with STZ. Taken together, cardiac dysfunction may be either associated with or due to subcellular abnormalities as a consequence of increase in plasma glucose and decrease in plasma insulin levels. Therefore this article aims to discuss the significance of subcellular defects with respect to Ca^2+^-handling in cardiomyocytes in diabetic heart. It also intends to describe hormonal imbalance that is associated with elevated plasma levels of catecholamines, angiotensin II and 5-hydroxytryptamine (5-HT) as well as increased fatty acid oxidation in diabetes and that leads to the generation of oxidative stress. It is the oxidative stress that is considered to be the main cause of subcellular abnormalities and altered Ca^2+^-handling in the cardiomyocytes and subsequent cardiac dysfunction in diabetes (Figure 2). Accordingly, different pharmacologic interventions that target increases in intracellular free Ca^2+^ concentration (verapamil), catecholamine action (propranolol), angiotensin II action (enalapril and losartan), 5-HT (sarpogrelate), metabolic defects (propionyl-L-carnitine) and oxidative stress (vitamin E) should be indicated in attenuating subcellular remodeling for improvement of cardiac function and therapy of diabetic cardiomyopathy [6,26]. 

## 2. Alterations in Cardiac Function, Metabolism and Ultrastructure

Metabolic and ultrastructural derangements associated with insulin deficiency and/or insulin resistance can be seen to play an important role in the development of cardiac dysfunction in chronic diabetes [6,7,8,32]. Furthermore, it is conceivable that altered cardiac metabolism may be due to increases in plasma levels of norepinephrine, angiotensin II and 5-HT during diabetes [6,33,34,35,36,37]. It should be mentioned that while hyperglycemia and hyperlipidemia occur in diabetes, cardiomyocyte glucose uptake is reduced, but uptake of free fatty acids (FFA) is increased [6,7,8]. Such a shift in substrate supply is considered to result in a metabolic disturbance in the diabetic heart. 

In view of the involvement of mitochondria in the metabolism of glucose and FFA and their main function to produce ATP, a defect in energy production due to an impairment of the electron transport system in these organelles can be seen to be associated with increased formation of oxyradicals and occurrence of oxidative stress. It should be mentioned that although plasma levels of both glucose and FFA are elevated in diabetic subjects, glucose metabolism is markedly reduced due to impaired glucose transport in cardiomyocytes [8,38,39]. Furthermore, glucose uptake in the diabetic heart has been shown to be reduced as a consequence of elevated FFA levels, and FFA uptake is increased in the diabetic heart [40,41,42]. Because these metabolic changes in the diabetic heart are seen at early stages of the disease, it has been suggested that this metabolic shift may be the primary event leading to the generation of oxidative stress in the diabetic heart and the development of diabetic cardiomyopathy [28]. 

Several studies have investigated the morphological appearance of hearts from control and experimental diabetic animals [3,26,43]. The most notable changes in hearts from diabetic animals were increases in glycogen and lipid droplets compared with controls. This was accompanied by a loss of cellular integrity, disruption of myofibrils and swelling of sarcotubules in the hearts of diabetic animals. Extensive vacuolization and sarcomere contracture have also been observed in the diabetic heart [43]. Although no changes in the nucleus or lysosomes in any of the groups were seen, the mitochondria appeared larger in a cross-section orientation of the muscle compared with a longitudinal orientation [43]. Furthermore, disorganization and clearing of mitochondrial matrix and swelling of mitochondria have also been observed in the diabetic heart. Such ultrastructural pathology has been linked to the cardiac functional abnormalities during the development of diabetic cardiomyopathy. 

## 3. Subcellular Defects in Ca^2+^-handling

An excessive amount of intracellular Ca^2+^ has been shown to produce cell damage and contractile failure [9,26,44], and subcellular Ca^2+^-handling abnormalities have been associated with cardiac dysfunction in diabetic cardiomyopathy. A depression in the activity of SL Na^+^-K^+^ ATPase in the diabetic heart has been suggested to promote the entry of Ca^2+^ [44,45,46] whereas the depressed SL Na^+^- Ca^2+^ exchanger activity can be seen to raise the intracellular concentration of Ca^2+^ [45,47,48]. The decrease in the SL Ca^2+^-pump activity has been reported to depress the extrusion of Ca^2+^ from the diabetic cardiomyocytes [45,48,49]. The increased activity of SL Ca^2+^/Mg^2+^-ecto ATPase in diabetic heart has been considered to promote the entry of Ca^2+^ [50] whereas the reduced SL Na^+^-H^+^ exchanger would favor the accumulation of H^+^ for the release of Ca^2+^ from the intracellular Ca^2+^-stores [10,51,52]. The decreased SL ATP-independent Ca^2+^-binding and L-type Ca^2+^-channel density were suggested to reduce Ca^2+^-influx into the diabetic heart [53]. It is pointed out that alterations in SL Ca^2+^-channels are biphasic in nature, where at initial stages of diabetes, these changes promote Ca^2+^-entry, but at later stages, the reduction in SL Ca^2+^-channel density could lead to depressed cardiac contractile activity [26,54]. 

Several investigators have observed defects in SR Ca^2+^-uptake and Ca^2+^-pump ATPase activities [3,26]; SR Ca^2+^-release activity was also depressed in hearts from diabetic animals [55,56,57]. Various mechanisms, which are known to regulate the Ca^2+^-transport activities in the SR membranes, were impaired in the diabetic heart [25,26,28,54]. Such alterations in the regulation of SR Ca^2+^-uptake and -release activities in the diabetic heart seems to occur due to changes in the phosphorylation/dephosphorylation status of both SR and SL Ca^2+^-cycling proteins [45,58,59,60]. Taken together, Ca^2+^-handling abnormalities in SR would tend to favor the occurrence of intracellular Ca^2+^-overload [61,62,63,64], and this view is consistent with our observations that treatment of diabetic animals with a Ca^2+^-antagonist, verapamil, attenuated defects in subcellular activities [43,65]. Table 3 shows the hemodynamic parameters, subcellular activities and general characteristics of control, diabetic and verapamil-treated STZ-induced diabetic animals. It can be seen that myofibrillar ATPase activity, SR Ca^2+^- uptake and SR Ca^2+^-stimulated ATPase activities were decreased in the diabetic heart as compared to control values. Chronic treatment of diabetic rats with verapamil resulted in improvement of cardiac function as well as altered myofibrillar ATPase activity and SR Ca^2+^-pump activities without affecting the plasma glucose/insulin levels. Accordingly, it was suggested that normalization of subcellular organelle function in diabetic cardiomyopathy by verapamil may be related to its effects in controlling the entry of Ca^2+^ into the cardiac cell [43]. 

## 4. Hormonal Imbalance and Modification of Subcellular Remodeling and Cardiac Dysfunction

In view of the activation of SNS and increased level of plasma catecholamines in early stages of diabetes [33,34], it has been indicated that an excessive amount of circulating catecholamines may induce subcellular remodeling in the diabetic heart [6,26,66]. Treatment of diabetic subjects with some β-adrenoceptor blockers was shown to have beneficial effects; treatment of diabetic animals with propranolol (75 mg/kg/day) attenuated diabetes-induced cardiac dysfunction without any changes in plasma glucose or insulin levels [6]. Furthermore, the depressed SL Na^+^-K^+^ ATPase, Na^+^-dependent Ca^2+^-uptake and ATP dependent Ca^2+^-uptake, as well as SR Ca^2+^-uptake and Ca^2+^-release activities and MF Ca^2+^-stimulated ATPase activity in diabetic hearts, were attenuated by propranolol treatment [6]. These observations indicate that attenuation of subcellular remodeling in the diabetic heart by β-adrenoceptor blockade is associated with improved cardiac contractile activity.

Several clinical and experimental studies have suggested that the renin–angiotensin system (RAS) is activated during the development of cardiac dysfunction in diabetes [49,67]. The activation of RAS has been considered to contribute to the subcellular remodeling and occurrence of diabetic cardiomyopathy [6,26]. In fact, it was demonstrated that the treatment of diabetic animals with both enalapril, an ACE inhibitor, or losartan, an angiotensin II type I receptor blocker, attenuated the diabetes-induced alterations in cardiac function without any correction of the hyperglycemia or hypoinsulinemia [49,67]. Furthermore, the depressed SL Na^+^-K^+^-ATPase, Na^+^-dependent Ca^2+^-uptake, and Ca^2+^-pump, as well as reduced SR Ca^2+^-release and Ca^2+^-pump activities and decreased MF Ca^2+^-ATPase activity in the diabetic heart, were also improved (Table 4). These results demonstrate that the beneficial effects of RAS blockade in diabetes and improved cardiac performance are related to attenuation of SL, SR and MF defects in the heart. Interestingly, both enalapril and losartan treatment of the diabetic animals reduced the elevated cardiac MDA levels, suggesting that both these agents may attenuate the extent of oxidative stress due to the activation of RAS in diabetes. 

Since there is an increase in platelet aggregation and the circulating levels of 5-HT have been reported to be elevated in diabetes [35,68], the blockade of 5-HT receptors was shown to attenuate diabetes-induced cardiovascular complications. Indeed, it was reported earlier that sarpogrelate, a 5-HT2A receptor antagonist, attenuates the changes in serum insulin, glucose and lipid levels in addition to improving alterations in blood pressure and cardiac performance in STZ-induced diabetic animals [68]. Although the exact mechanisms of beneficial action of sarpogrelate remain to be fully elucidated, particularly with respect to subcellular remodeling and Ca^2+^-handling, it was suggested that the improvement in cardiac function in response to sarpogrelate in diabetes is due to a facilitation of glucose transporter expression levels and possibly insulin production [68]. Taken together, it is evident that there occurs a hormonal imbalance in diabetes that contributes to subcellular remodeling and the pathogenesis of diabetic cardiomyopathy. Importantly, blockade of SNS, RAS and platelet aggregation was shown to improve cardiac function and appears to superimpose the beneficial effects of insulin treatment alone. 

## 5. Modification of Metabolic Defects and Cardiac Dysfunction

Diabetes-induced cardiac dysfunction and changes in SR Ca^2+^-pump and SL Na^+^-K^+^ ATPase activities were improved by treatment of animals with etomoxir, an inhibitor of carnitine palmitoyltransferase [69,70,71]. Attenuation of metabolic derangements in diabetic myocardium by propionyl-L-carnitine treatment was also observed to decrease changes in SR Ca^2+^-pump, SL Na^+^-K^+^ ATPase and SL Na^+^-Ca^2+^ exchange as well as cardiac function without any changes in SL Ca^2+^-pump or MF Ca^2+^-stimulated ATPase activities (Table 5) [72,73]. These studies support the view that metabolic abnormalities are a consequence of an excessive utilization of FFA by mitochondria [6,7,8]. 

Since oxidative stress, generated by metabolic derangement and hormonal imbalance, has been indicated to be a key component contributing to subcellular abnormalities and subsequent cardiac dysfunction in diabetes [6,37], we have examined the therapeutic value of vitamin E, a well-known lipophilic antioxidant molecule. It can be seen from Table 6 that diabetes-induced depressions of SL Na^+^-K^+^ ATPase, SL Na^+^-Ca^2+^ exchange, SR Ca^2+^-uptake, SR Ca^2+^-release as well as MF Ca^2+^-stimulated ATPase activities were attenuated by treatment with vitamin E [26]. Furthermore, a reduction in the diabetes-induced increases in MDA and conjugated diene levels, markers of oxidative stress, were associated with an improvement in cardiac function without affecting the plasma glucose or insulin levels upon treatment with vitamin E [26]. These lines of evidence clearly demonstrate that oxidative stress plays a critical role in eliciting subcellular defects and cardiac dysfunction in chronic diabetes.

## 6. Alternative Therapeutic Options

Although type I diabetes is generally characterized by hypoinsulinemia, whereas type II diabetes is characterized by hyperinsulinemia, it should be emphasized that similar alterations in cardiac function and subcellular organelles are known to occur in both of these forms of diabetes [6,7,13,24,25,26,27,28]. While this review has focused on the occurrence of cardiac dysfunction in insulin-dependent (type 1) diabetes, impaired insulin signaling has been suggested as a contributory mechanism in the pathogenesis of both types of diabetic cardiomyopathy [74]. In fact, it is now evident that several factors, including Ca^2+^-handling abnormalities, neurohormonal activation and oxidative stress, are considered as mechanisms for the occurrence of cardiac dysfunction as a consequence of insulin resistance or depressed insulin signaling [74]. Accordingly, several other therapeutic options have been reported. In this regard, the effects of insulin–glucose infusion on LV function in non-insulin-dependent diabetic patients have been investigated. Insulin induced an increase in LVEF after submaximal work in healthy and diabetic humans, but the increase in the diabetic patients was significantly lower [75]. It was suggested that the increase in exercise-LVEF in response to insulin is likely due to an enhancement of ventricular contractility. Interestingly, a correlation between LVEF and the index of insulin sensitivity was observed in diabetic patients [75]. 

While this review has described the role of activated SNS and RAS neurohormonal systems in the pathogenesis of diabetic cardiomyopathy, cardiomyocytes are known to also produce opioid peptides and receptors; particularly, β-endorphin is increased in the plasma of patients with congestive heart failure (CHF). Interestingly, the acute effects of β-endorphin infusion in patients with mild to moderate CHF have been observed to improve LVEF, reduce systemic vascular resistance and diminish neurohormonal activation; these changes were associated with stimulation of the GH/IGF-1 pathway [76]. It is thus conceivable that β-endorphin could represent a treatment option for improving cardiovascular function in diabetic patients with cardiac dysfunction. Of all the hypoglycemic agents in the pharmacological arsenal against diabetes, thiazolidinediones, particularly pigolitazone, as well as metformin, appear to have additional effects in ameliorating oxidative stress and thus are seen as highly beneficial in the prevention of insulin resistance and diabetes [77,78]. In fact, combination therapy with pioglitazone and metformin could be a viable option in the treatment of type 2 diabetes induced cardiovascular complications [78]. The effects of liraglutide, a glucagon-like peptide-1 analogue, on cardiac function, morphology and markers of oxidative stress have recently been examined in type 2 diabetic patients. It was observed that liraglutide improved arterial stiffness, LV strain, LV morphological changes and NT-proBNP by reducing oxidative stress in newly diagnosed type 2 diabetic patients [79]. From the aforementioned, it is evident that there are several characteristics and overlapping mechanisms that contribute to cardiac dysfunction in both insulin-dependent and non-insulin-dependent diabetes and that the occurrence of oxidative stress appears to be the key component in the pathogenesis of diabetic cardiomyopathy.

## 7. Conclusions and Therapeutic Implications

**Figure 3:**
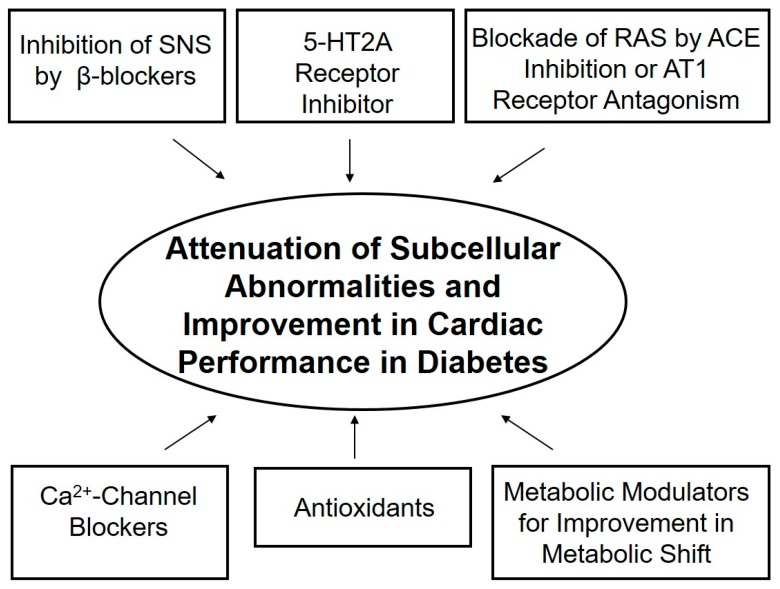
Interventions that may attenuate subcellular remodeling and improve cardiac performance in chronic diabetes.

The complexity of diabetic cardiomyopathy is confounded by the diverse range of mechanisms that are involved in the pathogenesis of cardiac dysfunction in diabetes. Increases in plasma levels of norepinephrine, angiotensin II and 5-HT have been observed at different stages of diabetes, which may contribute to the metabolic changes as well as functional alterations of the heart. In addition, depressed cardiac contractility in diabetes may also be due to subcellular remodeling in cardiomyocytes at the level of the SL, SR and MF. While the SL and SR defects in diabetic heart appear to result in Ca^2+^-handling abnormalities, defects of the MF would be more associated with insensitivity of MF to Ca^2+^. It is likely that Ca^2+^-handling defects, metabolic derangements and ultrastructural abnormalities may be a consequence of hormonal imbalance as well as occurrence of oxidative stress and intracellular Ca^2+^-overload. Pharmacological interventions that inhibit the SNS, RAS, 5-HT receptors and Ca^2+^-channels, as well as agents that can modulate fatty acid metabolism and those that can exert antioxidant actions, have been shown to partially prevent or normalize subcellular remodeling and cardiac dysfunction. Accordingly, it is suggested that a combination therapy with different interventions (as summarized in Figure 3) may prove beneficial for the treatment of heart dysfunction in diabetic cardiomyopathy.

## Figures and Tables

**Table 1:** Time course of changes in plasma glucose/insulin levels, cardiovascular function and subcellular activities in control and diabetic animals following alloxan injection.

Parameter	Control	Time after Alloxan Treatment (Weeks)				
		1 Week	2 Weeks	4 Weeks	8 Weeks	12 Weeks
Plasma glucose (mg/dl)	158 ± 15	523 ± 28 *	525 ± 19 *	495 ± 26 *	520 ± 24 *	512 ± 27 *
Plasma insulin (mU/dl)	2.95 ± 0.20	0.84 ± 0.17 *	0.91 ± 016 *	0.88 ± 0.14 *	0.82 ± 0.20 *	0.93 ± 0.18 *
Heart rate (beats/min)	383 ± 19	378 ± 15	368 ± 15	313 ± 21 *	280 ± 17 *	277 ± 14 *
MAP (mmHg)	123 ± 9	117 ± 2	117 ± 5	99 ± 4 *	93 ± 5 *	98 ± 7 *
LVEDP (mmHg)	2.4 ± 1.2	3.0 ± 1.1	3.5 ± 1.2	6.2 ± 1.1 *	6.1 ± 1.0 *	6.2 ± 1.4 *
LVSP (mmHg)	129 ± 7	130 ± 7	125 ± 8	110 ± 5 *	104 ± 4 *	107 ± 7 *
SL Na ^+^ -K^+^-ATPase (µmol Pi/mg/h)	24.6 ± 1.1	20.1 ± 0.9	16.8 ± 1.3 *	17.1 ± 0.7 *	16.5 ± 1.2 *	17.3 ± 1.1 *
SL Na^+^- Ca^2+^-exchanger (nmol Ca^2+^/mg/2 s)	5.01 ± 0.81	4.15 ± 0.81	2.80 ± 0.55 *	2.99 ± 0.67 *	2.75 ± 0.63 *	1.90 ± 0.80 *
SR Ca^2+^-stimulated ATPase (µmol Pi/mg/h)	10.07 ± 0.42	9.24 ± 0.30	6.95 ± 0.25 *	6.85 ± 0.33 *	6.04 ± 0.57 *	5.95 ± 0.39 *
SR Ca^2+^ uptake (nmol Ca^2+^/mg/min)	128 ± 10	119 ± 15	65 ± 7 *	50 ± 12 *	51 ± 9 *	64 ± 11 *
Myofibrillar Ca^2+^-stimulated ATPase (µmol Pi/mg/h)	12.7 ± 1.0	12.1 ± 0.9	8.0 ± 0.7 *	6.9 ± 0.7 *	6.6 ± 0.7 *	6.4 ± 0.7 *

Values are means ± S.E. of 6–15 animals in each group. MAP, mean arterial pressure; LVEDP, left ventricular end diastolic pressure; LVSP, left ventricular systolic pressure. * Statistically significantly different from respective control (*p* < 0.05). Data taken from our papers: Golfman et al. 1998 [29] and Golfman et al. 1999 [30]. Control values for all the parameters measured at the different time points were not significantly different and therefore were pooled and presented as mean ± S.E.

**Table 2:** Time-course of changes in hemodynamic parameters, plasma glucose and insulin profile of control and diabetic animals at different time points following streptozotocin (STZ) injection.

Parameter	Control	Time after STZ Treatment				
		15 Days	18 Days	21 Days	24 Days	27 Days
Plasma glucose (mg/dl)	130 ± 8	388 ± 21 *	425 ± 22 *	412 ± 20 *	448 ± 24 *	435 ± 24 *
Plasma insulin (µU/mL)	31.2 ± 1.7	12.4 ± 0.6 *	13.2 ± 0.5 *	12.7 ± 0.6 *	12.5 ± 0.7 *	13.1 ± 0.4 *
Heart rate (beats/min)	412 ± 5	424 ± 6	409 ± 6	401 ± 5	376 ± 5 *	362 ± 5 *
LVEDP (mmHg)	2.3 ± 1.4	4.7 ± 0.8	5.2 ± 1.6	8.6 ± 0.7 *	9.6 ± 0.9 *	9.8 ± 0.6 *
LVSP (mmHg)	150 ± 4	144 ± 4	138 ± 5	136 ± 3 *	131 ± 3 *	122 ± 3 *
SL Na^+^-K^+^-ATPase (µmol P_i_/mg/h)	25.2 ± 2.4	24.7 ± 3.1	21.2 ± 5.2	18.3 ± 3.4 *	17.2 ± 2.1 *	15.2 ± 1.9 *
SL Na^+^-Ca^2+^-exchanger (nmol Ca^2+^/mg/10 s)	21.4 ± 2.1	18.5 ± 2.2	17.6 ± 3.1	16.5 ± 3.2 *	16.0 ± 1.2 *	15.1 ± 1.1 *
SR Ca^2+^-stimulated ATPase (nmol Pi/mg/min)	158 ± 7	160 ± 4	149 ± 5	132 ± 6 *	124 ± 3 *	115 ± 6 *
SR Ca^2+^-uptake (nmol Ca^2+^/mg/min)	58 ± 5	52 ± 3	54 ± 5	44 ± 3 *	42 ± 3 *	39 ± 4 *
Myofibrillar Ca^2+^-stimulated ATPase (nmol Pi/mg/min)	192 ± 7	190 ± 14	182 ± 21	162 ± 16 *	149 ± 13 *	138 ± 12 *

Values are means ± S.E. of 10 different experiments. LVEDP, left ventricular end diastolic pressure; LVSP, left ventricular systolic pressure; * Statistically significantly different from control (*p* < 0.05). Data taken from our paper: Takeda et al. 1996 [31]. Control values for all the parameters measured at the different time points were not significantly different and therefore were pooled and presented as mean ± S.E.

**Table 3:** Plasma glucose/insulin levels, hemodynamic parameters and subcellular activities of control, diabetic and verapamil-treated diabetic animals.

Parameter	Control	Diabetes	Diabetes + Verapamil
Plasma glucose (mg/dl)	190 ± 5	706 ± 56 *	661 ± 9 *
Plasma insulin (mU/dl)	3.10 ± 0.25	0.90 ± 0.10 *	1.0 ± 0.15 *
Heart rate (beats/min)	357 ± 6	283 ± 7 *	334 ± 9 #
LVEDP (mmHg)	3.0 ± 2.0	19.0 ± 1.0 *	3.0 ± 0.7 #
LVSP (mmHg)	151 ± 2	123 ± 3 *	151 ± 3 #
+ dP/dt (mmHg/s)	6137 ± 176	4332 ± 226 *	5415 ± 90 #
− dP/dt (mmHg/s)	5415 ± 158	3610 ± 173 *	4693 ± 8
Myofibrillar Ca^2+^-stimulated ATPase (nmol Pi/mg/min)	148 ± 7	95 ± 5 *	134 ± 11 #
SR Ca^2+^-stimulated ATPase† (µmol Pi/mg/5 min)	0.89 ± 0.08	0.52 ± 0.05 *	0.92 ± 0.06 #
SR Ca^2+^-uptake† (nmol Ca^2+^/mg/min)	56 ± 6	30 ± 4 *	49 ± 6 #

Values are means ± S.E. of 8–12 experiments. Treatment with verapamil at 4 mg/kg was initiated in a randomly selected group of 4 wk diabetic animals for 4 wks. LVEDP, left ventricular end diastolic pressure; LVSP, left ventricular systolic pressure; +dP/dt, rate of pressure development; -dP/dt, rate of pressure decay. † In these experiments, verapamil treatment (4 mg/kg) was initiated 1 day after the induction of diabetes and continued for 8 wks. * Statistically significantly different from control (*p* < 0.05); # statistically significantly different from diabetic value. Data taken from our papers: Afzal et al. 1988 [43] and Afzal et al. 1989 [65].

**Table 4:** Glucose/insulin levels, hemodynamic parameters and subcellular activities of control, diabetic and enalapril- or losartan-treated diabetic animals.

	Control	Diabetes	Diabetes + Enalapril	Diabetes + Losartan
Plasma glucose (U/mL)	154 ± 9	489 ± 17 *	464 ± 12 *	471 ± 9 *
Plasma insulin (mg/mL)	29 ± 2	12 ± 2 *	13 ± 1 *	13 ± 1 #
+dP/dt (mmHg/s)	5840 ± 265	3780 ± 218 *	4764 ± 196 *#	4780 ± 225 *#
− dP/dt (mmHg/s)	5560 ± 164	3376 ± 187 *	4580 ± 208 *#	4548 ± 192 *#
LVSP (mmHg)	140 ± 12	85 ± 8 *	119 ± 8 #	116 ± 7 #
LVEDP (mmHg)	3.4 ± 0.2	3.9 ± 0.3	3.9 ± 0.2	4.1 ± 0.3
Myofibrillar Ca^2+^ -stimulated ATPase (nmol Pi/mg/5 min)	870 ± 21	524 ± 23 *	720 ± 26 *#	708 ± 16 *#
SL Na^+^-K^+^-ATPase (µmol P_i_/mg/h)	23.2 ± 3.5	13.1 ± 1.8 *	18.2 ± 1.6 #	18.3 ± 1.5 #
SL Na^+^- Ca^2+^-exchanger (nmol Ca^2+^/mg/10 s)	21.3 ± 1.2	12.1 ± 0.9 *	17.3 ± 1.2 *#	16.1 ± 1.5 *#
SR Ca^2+^- stimulated ATPase (nmol Pi/mg/5 min)	165 ± 7	115 ± 10 *	154 ± 5 #	153 ± 6 #
SR Ca^2+^-uptake (nmol Ca^2+^/mg/2 min)	62.7 ± 2.3	36.5 ± 3.1 *	50.3 ± 2.1 *#	53.4 ± 2.7 *#
SR Ca^2+^-release (nmol Ca^2+^/mg/15 s)	9.3 ± 0.4	5.8 ± 0.3 *	8.7 ± 0.5 #	8.5 ± 0.4 #
MDA (nmol/mg tissue lipids)	3.8 ± 0.13	7.1 ± 0.49 *	4.9 ± 0.5 *#	5.4 ± 0.6 *#

Values are means ± S.E. of 6–8 different experiments. Treatment of diabetic animals with enalapril (10 mg/kg, daily) and losartan (20 mg/kg, daily) by gastric tube was initiated 3 days after the induction of diabetes with streptozotocin (STZ) (65 mg/kg, i.v.) for 8 wks. +dP/dt, rate of pressure development; -dP/dt, rate of pressure decay; LVSP, left ventricular systolic pressure. * Statistically significantly different from control (*p* < 0.05); # statistically significantly different from diabetic (*p* < 0.05). Data taken from our papers: Liu et al. 2006 [49] and Machackova et al. 2004 [67].

**Table 5:** Glucose/insulin levels, hemodynamic parameters and subcellular activities of control, diabetic and propionyl-L-carnitine-treated diabetic animals.

	Control	Diabetes	Diabetes + PPLC
Plasma glucose (mg/dL)	137 ± 3	445 ± 18 *	215 ± 38 #
Plasma insulin (µU/mL)	55 ± 6	20 ± 2 *	23 ± 2 #
+dP/dt (mmHg/s)	5300 ± 150	4150 ± 200 *	5200 ± 175 #
-dP/dt (mmHg/s)	4600 ± 120	3700 ± 150 *	4800 ± 200 #
LVSP (mmHg)	160 ± 5	120 ± 7 *	165 ± 7 #
Myofibrillar Ca^2+^ -stimulated ATPase (nmol Pi/mg/5 min)	0.48 ± 0.015	0.33 ± 0.025 *	0.35 ± 0.021 *
SL Na^+^-K^+^-ATPase (µmol P_i_/mg/h)	19.8 ± 2.1	10.7 ± 1.6 *	15.7 ± 1.6 #
SL Na^+^-Ca^2+^-exchanger (nmol Ca^2+^/mg/30 s)	8.8 ± 1.6	3.2 ± 1.4 *	5.1 ± 1.2 *
SR Ca^2+^-stimulated ATPase (nmol Pi/mg/5 min)	814 ± 74	428 ± 50 *	784 ± 65 #
SR Ca^2+^-uptake (nmol Ca^2+^/mg/5 min)	224 ± 11	135 ± 15 *	205 ± 9 #

Values are means ± S.E. of four different experiments. Diabetes was induced by a single tail vein injection of STZ (55 mg/kg). PPLC treatment (3 g/kg, daily) was initiated at 3 days after induction of diabetes and continued for 6 wks. +dP/dt, rate of pressure development; -dP/dt, rate of pressure decay; LVSP, left ventricular systolic pressure; PPLC, propionyl-L-carnitine. * Statistically significantly different from control (*p* < 0.05); # statistically significantly different from diabetic (*p* < 0.05). Data taken from our paper: Dhalla et al. 1992 [73].

**Table 6:** Glucose/insulin levels, hemodynamic parameters and subcellular activities in control, STZ-induced diabetic and diabetic animals treated with vitamin E.

	Control	Diabetes	Diabetes + Vitamin E
Plasma glucose (mg/dl)	151 ± 8	487 ± 9 *	478 ± 9 *
Plasma insulin (µU/mL)	28 ± 2	11 ± 1 *	12 ± 1 *
+dP/dt (mmHg/s)	5722 ± 254	4210 ± 145 *	5450 ± 180 #
-dP/dt (mmHg/s)	5525 ± 129	4155 ± 135 *	5341 ± 182 #
Myofibrillar Ca^2+^ -stimulated ATPase (µmol Pi/mg/h)	11.6 ± 0.9	5.8 ± 0.5 *	8.4 ± 0.4 #
SL Na^+^-K^+^-ATPase (µmol Pi/mg/h)	24.7 ± 6	16.1 ± 2.7 *	23.6 ± 2.9 #
SL Na^+^- Ca^2+^-exchanger (nmol Ca^2+^/mg/2 s)	3.9 ± 0.2	2.1 ± 0.2 *	3.7 ± 0.4 #
SR Ca^2+^-release (nmol Ca^2+^/mg/3 min)	20.5 ± 2.1	10.2 ± 1.3 *	19.1 ± 1.8 #
SR Ca^2+^-uptake (nmol Ca^2+^/mg/min)	79.5 ± 7.1	43.7 ± 4.3 *	62.8 ± 3.2 *#
MDA (nmol/mg tissue lipids)	4.2 ± 0.3	6.9 ± 0.4 *	4.3 ± 0.5 #
Conjugated dienes (nmol/mg tissue lipids)	39.6 ± 3.2	68.3 ± 7.1 *	46.7 ± 5.4 #

Values are means ± S.E. of six experiments. Treatment with vitamin E (25 mg/kg/day, i.p.) was started 24 h after inducing diabetes with STZ. * Statistically significantly different from control (*p* < 0.05); # statistically significantly different from diabetic value. Data taken from our paper: Dhalla et al. 1998 [26].
